# Insight into thermophiles and their wide-spectrum applications

**DOI:** 10.1007/s13205-016-0368-z

**Published:** 2016-02-23

**Authors:** Ridhi Mehta, Paavan Singhal, Hardeep Singh, Dhanashree Damle, Anil K. Sharma

**Affiliations:** 1 Department of Biotechnology, M.M. University, Mullana, Ambala, Haryana 133207 India; 2 Department of Orthodontics, M.M.Institute of Dental Sciences & Research, M.M. University, Mullana, Ambala, Haryana 133207 India

**Keywords:** Thermophilic microorganisms, Thermostable enzymes, Heat tolerance and biomass

## Abstract

The deconstruction of biomass is a pivotal process for the manufacture of target products using microbial cells and their enzymes. But the enzymes that possess a significant role in the breakdown of biomass remain relatively unexplored. Thermophilic microorganisms are of special interest as a source of novel thermostable enzymes. Many thermophilic microorganisms possess properties suitable for biotechnological and commercial use. There is, indeed, a considerable demand for a new generation of stable enzymes that are able to withstand severe conditions in industrial processes by replacing or supplementing traditional chemical processes. This manuscript reviews the pertinent role of thermophilic microorganisms as a source for production of thermostable enzymes, factors afftecting them, recent patents on thermophiles and moreso their wide spectrum applications for commercial and biotechnological use.

## Introduction

Organisms with an optimum temperature for growth between 60 and 80 °C are generally designated as thermophiles, while those growing optimally above 80 °C are referred to as hyperthermophiles (Santos and Da Costa 2002). Thermophilic bacteria are microbes that mostly inhabit hot springs, live and survive in temperatures above 70 °C. As a consequence of growth at high temperatures and unique macromolecular properties, thermophiles can possess high metabolism, physically and chemically stable enzymes and lower growth but higher end product yields than similar mesophilic species (Haki and Rakshit 2003) (Tables 1, 2).

**Table 1:** Thermophilic enzymes and their potential roles

Microorganisms	Enzymes	Temperature of activity	Applications	References
*Pyrococcus woesei*	alpha-Amylases	Topt. = 100 °C	Sugar industry and starch processing	Alqueres et al. (2007)
*Thermococcus profundus* DT5432	alpha-Amylases	Topt. = 80 °C	Sugar industry and starch processing	Eichler (2001), Antranikian et al. (2005)
*Staphylothermus marines*	Pullulanases	Topt. = 90–105 °C	Sugar industry and starch processing	Eichler (2001), Antranikian et al. (2005)
*Thermoplasma acidophilum*	Glucoamylases	Topt. = 90 °C	Sugar industry and starch processing	Eichler (2001), Antranikian et al. (2005)
*Pyrococcus woesei*	β-Galactosidases	Topt. = 93 °C	Production of milk with low lactose content	Dabrowski et al. (1998)
*Pyrococcus furiosus Sulfolobales sp.*	Cellulases	Topt. = 103 °C	Production of alcohol, fruit industry	Antranikian et al. (2005)
*Pyrodictium abyssi*	Xylanases	Topt. = 100–110 °C	Paper industry–bleaching of pulp	Egorova and Antranikian (2005), Eichler (2001)
*Humicola lanuginosa* strain Y-38	Lipases	Topt. = 65 °C	Laundry detergents	Arima et al. (1972)
*Myceliophthora thermophila*	Laccases	Topt. = 60 °C	Polymerization of phenolic compounds to humic substances	Chefetz et al. (1998)
*Myceliophthora thermophila*	Phytases	Topt. = 42–45 °C	Animal feed	Wyss et al. (1999)
*Penicillium duponti*	glucose-6-phosphate dehydrogenase	Topt. = 50 °C	Generation of NADPH for biosynthetic reactions	Broad and Shepherd (1970)
*Bacillus lichniformis*	Alcalase	Topt. = 60 °C	Component of protein-fortified soft drinks and dietetic food, helps in protein recovery from meat, fish and crustacean shell waste	Synowiecki (2008)

**Table 2:** Recent patents on thermophiles and their potential applications

S. no	Topic	Patent number and date	Application	References
1	Single step bioconversion of lignocellulosic biomass to biofuels using extreme thermophilic bacteria	US2014/0363869 A1 December 11, 2014	Bioconversion of lignocellulosic biomass to biofuels	Curvers et al. (2014)
2	Thermophilic bacterium and uses of extracellular proteins therefrom	US 8828238 B2 September 9, 2014	Excellent metal ion binding ability	Han et al. (2014)
3	Fermentation of moderately thermophilic *Bacilli* on sucrose	US 8,663,954 B2 March 4, 2014	Genetic modification of moderately thermophilic *Bacillus* strain to utilise sucrose as a carbon source	Van Kranenburg et al. (2014)
4	Bioremediation of persistent organic pollutants using thermophilic bacteria	US 2014/0042087 A1 February 13, 2014	Degradation of organic pollutants	O’Driscoll et al. (2014)
5	Phytase-producing bacteria, phytase and production method of phytase	US 6,180,390 B1 January 30, 2001	Role in animal feeding, environmental protection, human nutrition and health and industrial applications.	Chu et al. (2001)
6	Process for producing modified microorganisms for oil treatment at high temperatures, pressures and salinity	US 5492828A February 20, 1996	Used in microbial enhanced oil recovery	Eugene et al. (1996)

Natural environments for anaerobic thermophiles range from terrestrial volcanic sites (including solfatara fields) with temperatures slightly above ambient temperature, to submarine hydrothermal systems (sediments, submarine volcanoes, fumaroles and vents) with temperatures exceeding 300 °C, subterranean sites such as oil reservoirs, and solar heated surface soils with temperatures up to 65 °C. There are also human-made hot environments such as compost piles (usually around 60–70 °C but as high as 100 °C) slag heaps, industrial processes and water heaters (Oshima and Moriya 2008).

The ubiquitous nature of the thermophiles is attested to by the great variety of sources from which they have been isolated from freshly fallen snow (Golikowa 1926) to the sands of the Sahara Desert (Negre 1913). They have been found to occur in the air (Sames 1900), the soil of temperate (Blau 1906; Gilbert 1904; Sames 1900) and tropical (De Kruyff, 1910) regions, salt (MacFadyen and Blaxall 1896) and fresh water, both cold (Tirelli 1907; Catterina 1904) and thermal (Georgevitch 1910a, b; Falcioni 1907; Benignetm 1905; Setchell 1903).

Factors affecting heat tolerance of thermophilic organisms are as follows:

1. Permeability: cell membranes effectively function as a permeability barrier, controlling the in-flow and out-flow of low-molecular weight compounds. The permeability of fatty acyl ester lipid membranes is highly temperature dependent and their phase-transition temperature is dependent on the fatty acid composition, so when the growth temperature shifts, the fatty acid composition of membrane lipids is quickly regulated (Koga 2012).
2. Chemical stability: thermophilic organisms are able to grow at high temperature due to the chemical stability of their membrane lipids (Koga 2012).
3. Temperature: lipids that increase in proportion to an increase in growth temperature may be designated as “thermophilic lipids.” In the extremely thermophilic environment, methanoarchaea *Methanocaldococcus jannaschii* have been reported. When the growth temperature increases from 45 to 65 °C, the diether lipids (archaeolbased lipids) decrease from 80 to 20 %, while the standard caldarchaeol-based and cyclic archaeol-based lipids increase from 10 to 40 %, respectively (Sprott et al. 1991).
4. G+C content: rRNA and tRNA molecules of thermophilic bacteria have higher G+C contents than mesophiles (Galtier and Lobry 1997). Because the GC base pair forms more hydrogen bonds than the AT base pair, higher G+C contents in the double-stranded stem region improves thermostability of the RNA molecules (Lao and Forsdyke 2000; Paz et al. 2004).
5. Proteins: the surface regions of thermophilic proteins have fewer (non-charged) polar amino acids and more charged amino acids, and these charged residues result in an increased number of intramolecular salt bridges (Thompson and Eisenberg 1999).

The ability of microorganisms to survive under harsh conditions has prompted researchers to study these organisms to better understand their characteristics and eventually utilize them in various applications. Further insight into thermophilic microorganisms has been highlighted through this review article as thermophiles possess enumerable properties suitable for biotechnological and commercial applications.

## Biotechnological applications of thermophiles

**Fig. 1:**
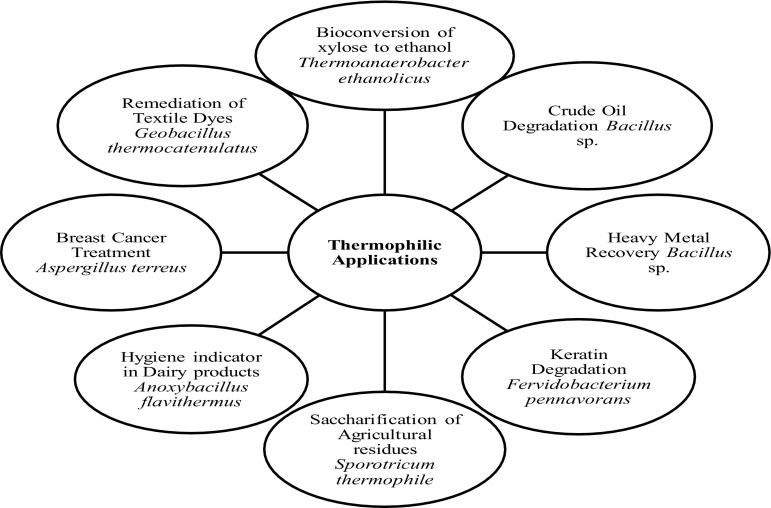
Various applications of thermophilic microorganisms

Thermophiles have shown tremendous promise in terms of their applications in modern biotechnology. Some of the high end applications of these thermophiles have been elucidated below (Fig. 1).

### Bioconversion of lignocellulose to hydrogen

Although many reported microorganisms possess the capability of cellulose hydrolysis or hydrogen production (H_2_), no conclusive research has been able to clarify that both of these capabilities are possessed in a single microorganism. Hot springs are a potential source for thermophilic hydrogen (H_2_) and ethanol producing micro-organisms. Compared with mesophiles, thermophiles are thought to be more robust for cellulose degradation and hydrogen production. In particular, the rate of cellulolysis is presumably more rapid at elevated temperatures (Wiegel and Ljungdahl 1986; Blumer-Schuette et al. 2008). As a result, thermophilic microorganisms isolated from various environments are an attractive prospect for cellulolytic biohydrogen production (CBP) from complex lignocellulosic biomass. The co-cultures of thermophilic cellulolytic bacterium *Clostridium thermocellum* with non-cellulolytic thermophilic anaerobic bacteria and the extremely thermophilic cellulolytic bacterium *Caldicellulosiruptor saccharolyticus* have been used for CBP-based hydrogen production (Liu et al. 2008; Ivanova et al. 2009). Several species of genus Thermoanaerobacterium including *T*. *thermosaccharolyticum*, *T*. *polysaccharolyticum*, *T*. *zeae*, *T*. *lactoethylicum*, *T*. *aotearoense*, and *T*. *saccharolyticum* possess the capability to utilize various macromolecules accompanied by H_2_ production (Ganghofer et al. 1998; Hoster et al. 2001; O-Thong et al. 2008; Ren et al. 2008; Cann et al. 2001; Ueno et al. 2001).

Several anaerobic thermophiles have been shown to utilize cellulose, including *Clostridium thermocellum*, *Clostridium* *straminisolvens*, *Clostridium stercorarium*, *Caldicellulosiruptor* *saccharolyticus*, and *Caldicellulosiruptor obsidiansis* (Freier et al. 1988; Hamilton-Brehm et al. 2010; Kato et al. 2004; Madden 1983; Rainey et al. 1994; Wiegel and Dykstra 1984).

Hydrogen may be the fuel of the future once hydrogen fuel cells for propelling cars are perfected (McAlister 1998). When oxygen and hydrogen are combined in a fuel cell, they provide electricity and a little heat, giving water as the only waste product. The hydrogen car will be clean because it will not discharge nitrogen oxides and carbon dioxide. Hydrogen can be obtained cheaply using special enzymes (extremozymes) by the transformation of cellulose into glucose sugar, then converting the glucose product and its byproduct, gluconic acid into hydrogen (Woodward et al. 2000).

The extremely thermophilic bacterium *C*. *owensensis* has comprehensive hemicellulase and cellulase system. The enzymes of *C*. *owensensis* had high ability for degrading the hemicellulose of native lignocellulosic biomass. High temperature pre-hydrolysis on native lignocellulosic biomass by the extra-enzyme of *C*. *owensensis* could greatly improve the glucan conversion rate, making almost the same contribution as steam-exploded pretreatment (Peng et al. 2015).

### Conversion of glycerol to lactate

Bioprospecting efforts for exploring novel biocatalytic molecules with unique properties have inspired the design and construction of a wider variety of artificial metabolic pathways (Bond-Watts et al. 2011). Employment of enzymes derived from thermophiles and hyperthermophiles enables the simple preparation of catalytic modules with excellent selectivity and thermal stability (Ye et al. 2012; Ninh et al. 2015). These excellent stabilities of thermophilic enzymes allow greater flexibility in the operational conditions of in vitro bioconversion systems. Generally, 10 kg of crude glycerol, which is the primary byproduct of the biodiesel industry, is released for every 100 kg of biodiesel and the growing production of biodiesel has resulted in a worldwide surplus of crude glycerol (Nguyen et al. 2013). An artificial in vitro metabolic pathway for the conversion of glycerol to lactate has been constructed. The in vitro pathway consisted of nine thermophilic and hyperthermophilic enzymes and designed to balance the intrapathway consumption and regeneration of cofactors (Okano et al. 2010).

### Conversion of d-xylose into ethanol

Thermophilic anaerobic bacteria could be promising candidates for conversion of hemicellulose or its monomers (xylose, arabinose, mannose and galactose) into ethanol with a satisfactory yield and productivity. A number of thermophilic enrichment cultures, and new isolates of thermophilic anaerobic bacterial strains growing optimally at 70–80 °C for their ethanol production from d-xylose have been isolated from hot springs, paper pulp mills and brewery waste water. The species investigated so far are *Thermoanaerobacter ethanolicus* (Wiegel and Ljungdahl 1981; Kannan and Mutharasan 1985), *Clostridium thermocellum* (Viljoen et al. 1926; McBee 1954; Ng et al. 1981), *Clostridium thermohydrosulfuricum* (Cook and Morgan 1994; Wiegel et al. 1979; Do¨nmez and O¨zcelik 1992) (reclassified as *Thermoanaerobacter thermohydrosulfuricus*) (Lee et al. 1993), *Thermoanaerobium brockii* (Zeikus et al. 1979; Ben-Bassat et al. 1981) (reclassified as *Thermoanaerobacter brockii*) (Lee et al. 1993), *Clostridium thermosaccharolyticum* (McClung 1935; Mistry and Cooney 1989) (reclassified as *Thermoanaerobacterium thermosaccharolyticum*) (Collins et al. 1994) and *Thermoanaerobacterium saccharolyticum* B6A (Lee et al. 1993; Weimer 1985). To obtain a viable bio-ethanol production, all the carbohydrate constituents of lignocellulosic biomass need to be converted into ethanol (Wright 1988; Lynd 1990; Von Sivers and Zacchi 1995). Xylan is mainly composed of D-xylose and it has been reported that most of the isolates produced ethanol as the main end fermentation product from both xylose and xylan (Sommer et al. 2004). Among the advantages, thermophiles have broad substrate spectra and can degrade both hexoses and pentoses simultaneously; some thermophiles natively degrade complex carbohydrates; they operate at temperatures that minimize contamination risk of mesophiles. Additionally, recent advances have improved ethanol yields by using genetic engineering, often by knocking out metabolic pathways to other end products.This has minimized the perceived advantage of mesophiles over thermophiles considerably, although no large scale bioethanol plants with genetically modified microbes are currently operating (Scully and Orlygsson 2015).

### Biodegradation of petroleum hydrocarbons

Thermophiles have also been utilized for the microbial degradation of crude oil and refined petroleum pollutants. Foght and McFarlane (1999) studied the growth of extremophiles on petroleum hydrocarbon. Some potential applications are related to molecular genetics of polycyclic aromatic hydrocarbon degradation by bacteria. Also the factors that control degradation and methods to enhance the ability of bacteria to degrade such pollutants in the environment have been studied. April et al. (2000) studied the process of crude oil degradation by mixed populations, pure cultures, and genetic mutants. They demonstrated the loss of parent compounds and analyzed the products of bacterial attack on crude oils using gas chromatography, mass spectrometry. The use of thermophiles for biodegradation of hydrocarbons with low water solubility is of interest, as solubility and bioavailability, are enhanced at elevated temperatures. Thermophiles, predominantly *bacilli*, possess a significant potential for the degradation of environmental pollutants, including all major classes. Indigenous thermophilic hydrocarbon degraders are of special significance for the bioremediation of oil-polluted desert soil (Margesin and Schinner 2001).

### Recovery of heavy metals

As a result of increasing industrial activities, heavy metal contamination is a problem. Microorganisms can interact with heavy metals in a variety of ways that result in decreased metal mobility and solubility. The metal and sulfate-reducing bacteria have suitable physiology for metal precipitation and immobilization. The activities of these microbes provide metabolic products such as iron and hydrogen sulphide, which lead to mineral formation. These minerals can react with heavy metals, resulting in precipitation and hence detoxification (Chalaal and Islam 2001). In order to understand the removal of such types of toxins, Chalaal and Islam (2001) used two strains of thermophilic bacteria belonging to the *Bacillus* family, isolated from hot water stream, to remove strontium from aqueous stream systems. These bacteria were able to concentrate strontium in one side of a two-compartment bioreactor. Immobilization of heavy metals using sulphide-producing microorganisms has been reported as an effective means of treating some metal-contaminated sites (Crawford and Crawford 1996).

### Remediation of textile dyes

Laccase enzyme purified from thermophile, *Geobacillus thermocatenulatus* MS5 is of very higher catalytic activity and are economic, highly stable at different temperatures and pH levels and can be used widely and effectively in the removal of the dyes that cause environmental pollution. Verma and Shirkot investigated the purified laccase enzyme for the removal of some dyes used in industry i.e., Remazole Brilliant Blue R (RBBR), Indigo carmine, Congo red, Brilliant green and Bromophenol blue. In case of Indigo carmine and congo red dye, 99 % of decolorization occured after 48 h of incubation, followed by RBBR dye, Bromophenol Blue and Brilliant Green i.e., 98, 70 and 60 % respectively (Verma and Shirkot 2014).

Thermophilic lignolytic fungal cultures were isolated from soil/digested slurry/plant debris and were subjected for acclimatization to Remazol Brilliant Blue (RBB) at 0.05 % concentration, in the malt extract broth (MEB). The results suggested the isolates as a useful tool for degradation of reactive dyes (Sahni and Gupta 2014).

### Saccharification of agricultural residues

*Sporotricum thermophile* LAR5 is an excellent fungal isolate having an ability to utilize crude agriculture based materials as carbon and nitrogen sources to produce significant cellulase titre. Cellulase possesses desirable properties from industrial application point of view such as activity and stability over broad pH range and high temperatures and good saccharification ability on acid-pretreated rice straw. It has been reported that considerable sugars are produced by enzymatic hydrolysis of acid-pretreated solids (3.5, 5.7, 7.9, 7.7 micromoles/ml from 1, 3, 5 and 7 % acid-pretreated solids, respectively) using the *S*. *thermophile* LAR5 cellulase (Bajaj et al. 2014). Recombinant *S*. *thermophile* cellulase shows potential to hydrolyze variety of cellulosic substrates with a peculiarity that presence of lignin in various substrates enhances the degree of saccharification (Dimarogona et al. 2012).

### Thermophilic bacilli in dairy processing

Thermophilic bacilli are used as hygiene indicators of processed product, within the dairy processing context. This is because of the ability of these strains to form endospores and biofilms. The thermophilic bacilli, such as *Anoxybacillus flavithermus* and *Geobacillus* spp., are an important group of contaminants in the dairy industry. Although these bacilli are generally not pathogenic, their presence in dairy products is an indicator of poor hygiene and high numbers are unacceptable to customers. In addition, their growth may result in milk product defects caused by the production of acids or enzymes, potentially leading to off-flavors (Burgess et al. 2010). Many strains of genera *Lactobacillus* and *Bifidobacterium*, as well as some enterococci and yeasts, have been shown to possess probiotic properties with potential for prophylaxis and treatment of a range of gastrointestinal disorders (Varankovich et al. 2015).

### Keratin degradation

A novel thermophilic bacterium, *Fervidobacterium pennavorans*, belonging to the *Thermotogales* order, isolated from hot springs of Azores island, grows optimally at 70 °C and pH 6.5. It is the first known thermophile that is able to degrade native feathers at high temperatures. With the help of these enzymes, feathers could be converted to defined products such as the rare amino acids, serine, cysteine and proline (Friedrich and Antranikian 1996).

### Cancer treatment

Asperjinone, a nor-neolignan, and Terrein, a suppressor of ABCG2-expressing breast cancer cells were isolated from thermophile *Aspergillus terreus,* which can restore drug sensitivity and could be the key to improve breast cancer therapeutics. Terrein, displayed strong cytotoxicity against breast cancer MCF-7 cells. Treatment with terrein significantly suppressed growth of ABCG2-expressing breast cancer cells. This suppressive effect was achieved by inducing apoptosis via activating the caspase-7 pathway and inhibiting the Akt signaling pathway, which led to a decrease in ABCG2-expressing cells and a reduction in the side-population phenotype (Liao et al. 2012). Conventional chemotherapeutic agents are usually non specific towards cancerous cells and inhibit the progression of any dividing cells. The therapeutic potential of antitumor drugs is seriously limited by the manifestation of serious side effects and drug resistance. So there is a need of agents that are more effective, more selective and may not cause drug resistance. According to Patent no. WO 2006/053445 A1, an invention is disclosed, whereby a composition of bacteriocin derived from lactic acid bacteria and a carrier can be used for inhibiting proliferation of cancerous cells (Mehta et al. 2013).

## Conclusions

The increasing number of patents indicates that there is a growing interest in the commercial applications of thermophiles. The demand for thermostable enzymes has increased tremendously in the past few years. Since only a very few species from this group of microorganisms have been isolated till date, there seems to be a large number of hyperthermophilic catalysts with unique properties awaiting discovery.
